# Combining the new injury severity score with an anatomical polytrauma injury variable predicts mortality better than the new injury severity score and the injury severity score: a retrospective cohort study

**DOI:** 10.1186/s13049-016-0215-6

**Published:** 2016-03-08

**Authors:** Ting Hway Wong, Gita Krishnaswamy, Nivedita Vikas Nadkarni, Hai V. Nguyen, Gek Hsiang Lim, Dianne Carrol Tan Bautista, Ming Terk Chiu, Khuan Yew Chow, Marcus Eng Hock Ong

**Affiliations:** Department of General Surgery, Singapore General Hospital, Outram Road, Singapore, 169608 Republic of Singapore; Duke-National University of Singapore, Singapore, Singapore; Health Promotion Board, Singapore, Singapore; Tan Tock Seng Hospital, Singapore, Singapore; Department of Emergency medicine, Singapore General Hospital, Singapore, Singapore

**Keywords:** Trauma, Injury severity score, New injury severity score, Polytrauma, Mortality, Outcome prediction

## Abstract

**Background:**

Anatomy-based injury severity scores are commonly used with physiological scores for reporting severity of injury in a standardized manner. However, there is lack of consensus on choice of scoring system, with the commonly used injury severity score (ISS) performing poorly for certain sub-groups, eg head-injured patients. We hypothesized that adding a dichotomous variable for polytrauma (yes/no for Abbreviated Injury Scale (AIS) scores of 3 or more in at least two body regions) to the New Injury Severity Score (NISS) would improve the prediction of in-hospital mortality in injured patients, including head-injured patients—a subgroup that has a disproportionately high mortality. Our secondary hypothesis was that the ISS over-estimates the risk of death in polytrauma patients, while the NISS under-estimates it.

**Methods:**

Univariate and multivariable analysis was performed on retrospective cohort data of blunt injured patients aged 18 and over with an ISS over 9 from the Singapore National Trauma Registry from 2011–2013. Model diagnostics were tested using discrimination (c-statistic) and calibration (Hosmer-Lemeshow goodness-of-fit statistic). All models included age, gender, and comorbidities.

**Results:**

Our results showed that the polytrauma and NISS model outperformed the other models (polytrauma and ISS, NISS alone or ISS alone) in predicting 30-day and in-hospital mortality. The NISS underestimated the risk of death for patients with polytrauma, while the ISS overestimated the risk of death for these patients.

When used together with the NISS and polytrauma, categorical variables for deranged physiology (systolic blood pressure of 90 mmHg or less, GCS of 8 or less) outperformed the traditional ‘ISS and RTS (Revised Trauma Score)’ model, with a c-statistic of greater than 0.90. This could be useful in cases when the RTS cannot be scored due to missing respiratory rate.

**Discussion:**

The NISS and polytrauma model is superior to current scores for prediction of 30-day and in-hospital mortality. We propose that this score replace the ISS or NISS in institutions using AIS-based scores.

**Conclusions:**

Adding polytrauma to the NISS or ISS improves prediction of 30-day mortality. The superiority of the NISS or ISS depends on the proportion of polytrauma and head-injured patients in the study population.

## Background

Scoring systems to assess injury severity are used for institutional benchmarking, trauma system audit, and prediction of mortality. Age, sex, anatomical severity, physiological severity, and mechanisms of injury have been proposed as the basic covariates in predicting trauma outcomes [1]. In ageing populations, comorbidities are also important predictors for predicting trauma outcomes [2, 3]. Most consensus-based anatomical injury severity scores are based on the Abbreviated Injury Scale (AIS) of the Association of the Advancement of Automotive Medicine (AAAM), of which the most widely used severity scores are the Injury Severity Score (ISS) and the New Injury Severity Score (NISS) [4–6]. The NISS is the sum of the squares of the three highest injury scores regardless of body region [5], while the ISS is the sum of the squares of the injury scores in the three most severely injured body regions [4].

There have been many studies comparing the ISS and the NISS [6–15]. While most studies on blunt trauma patients showed the NISS to be superior to ISS, one large study showed the ISS to be superior [13], and another large study showed that patients with their two worst injuries in different body regions had worse outcomes than patients with their two worst injuries in the same body region [16]. At the time of these studies, the definition of polytrauma had not been fully established, and the proportion of polytrauma patients in these study populations was not reported.

Recent papers on polytrauma define anatomical polytrauma as having AIS scores of 3 or more in at least two body regions [17–19]. These patients would also meet current definitions of severe injury, an ISS or NISS of 16 or more [17, 18, 20, 21]. Advances in trauma systems have improved outcomes for the severely injured, and it is likely that polytrauma patients have benefited from the enhanced coordination of care [22–25].

The Berlin polytrauma definition proposed two additional physiological variables (systolic blood pressure (SBP) and GCS), two biochemical variables (acidosis or coagulopathy), and one age criterion (70 years of age). This was in response to the criticism that the anatomical definition of polytrauma alone did not sufficiently account for the increase in mortality [26]. These variables were proposed based on an empirical data that showed increased mortality if any of these criteria were met. Hence, the blood pressure or GCS physiological criteria in the Berlin polytrauma definition could be useful in addressing the problem of missing RTS values, where missing respiratory rate is the major cause for missing RTS [8].

Our study hypothesis was that adding a dichotomous variable for anatomical polytrauma (yes/no for AIS and scores of 3 or more in at least two body regions) to the NISS model, would be superior to a model with both ISS and polytrauma, and to the NISS or ISS alone. For brevity, “polytrauma”, when mentioned in this paper, will refer to this anatomical definition alone, unless otherwise specified. We tested a number of hypotheses:

i) For prediction of mortality in blunt trauma patients, the model combining NISS and polytrauma outperforms ISS and polytrauma, NISS alone, and ISS alone;
ii) Adding the polytrauma variable to the NISS would improve outcome prediction more than adding it to ISS;
iii) Compared to the ISS, the NISS underestimates the risk of mortality for patients with polytrauma, while the ISS overestimates mortality in polytrauma patients.

## Methods

### Study setting

Singapore is an Asian urban country with a long life expectancy and a centralized pre-hospital ambulance system [27, 28]. It has a land area of 719.1 square kilometres and a population of 5.5 million. It has a mixed public healthcare system [29].

### Data source and data collection

Data used for this research is from the Singapore National Trauma Registry (NTR), set up in 2011 [30]. Data in the NTR is collected from all public hospitals receiving Singapore Civil Defence Force (SCDF) ambulances (private hospitals are excluded from the NTR). Coding and data collection is performed by teams of trained trauma data coordinators (TCs) based at each public hospital, with data cleaning, data completeness, data validation, and inter-rater audits (which covers data accuracy and reliability) performed annually. Quarterly reviews of data capture problems are performed by a central pool based at the National Registry of Diseases Office (NRDO). Once a trauma patient is identified by its ICD9 code in the emergency department, data coding personnel will check that the patient meets the inclusion criteria. Some fields are then automatically included in the registry by data capture from electronic medical records and all fields are checked by TCs. All TCs are trained in coding of the AIS, capturing the physiological variables from medical records for the calculation of the RTS [31] and the calculation of ISS. Death date and cause of death post-discharge are captured at quarterly intervals from the registry of births and deaths.

### Study population

The study sample consists of patients aged 18 and above presenting to the emergency departments of public hospitals with any injury with the diagnostic codes 800 – 959.9 defined in the International Classification of Diseases, 9th Revision, Clinical Modification (ICD-9-CM) [32], with an ISS of 9 or more. Isolated same-level fall hip fractures in the over 65 were excluded as detailed comorbidity information was not routinely collected for these patients by the NTR at the time of study. Both Singapore residents and non-residents were included in the study. Since penetrating injuries constituted less than 1 % of the study population, they were excluded from this study. Burns, drownings, and hangings were also excluded.

### Study design

Retrospective data from the NTR from January 2011 to December 2013 was matched with death registry data to calculate 30-day mortality.

### Study outcomes

Thirty-day mortality was the primary outcome measure [21]. Sensitivity analysis was performed using in-hospital mortality.

### Covariables

#### Age

We defined adults as aged over 18. Age was analyzed in bands, the youngest band aged 18–44, followed by 10-year bands from age 45, the highest age band as age 85 and over, with the 65–74 year age band split into 5-year bands, with the goal of including all the commonly used age cut-offs in the literature [17, 33–35]. Sensitivity analysis using age as a continuous variable was performed.

#### Injury severity measures

The ISS, the NISS, and polytrauma were derived from the AIS scores for patients. Physiological variables were the RTS, and in alternative models, RTS was replaced by categorical variables for GCS of 8 or less, systolic blood pressure of 90 mmHg or less. At the time of the study, laboratory parameters were not routinely included in the registry. Hence, coagulopathy and acidosis were not included in our models.

#### Presence of comorbidities

The Charlson Comorbidity Index was the primary measure of comorbidities [36]. This was derived from patient medical histories entered by the TCs as ICD-9 codes, and then re-classified by the Clinical Classifications Software tool by the Agency for Healthcare Research and Quality (AHRQ) [37].

### Potential bias

The NTR would not capture injuries for which the SCDF ambulance was not called and for whom a private ambulance was called to transport the patient directly to a private hospital or direct ward admissions bypassing the emergency department. This would have a minor effect on the capture of moderate injuries as SCDF usage is high [27].

### Statistical methods

Patient characteristics at baseline were summarized by mean (with standard deviation) or median (with inter-quartile range) or frequency (%) as appropriate. Chi-square tests and Fisher’s exact test were performed to evaluate associations between the outcomes of interest and other categorical predictors of interest. Multivariable logistic regression was used to analyze in-hospital mortality, adjusting for ISS, NISS, RTS, systolic blood pressure 90 mmHg or less, GCS 8 or less, polytrauma, race, residency, and comorbidities.

Those predictors that were significant in the univariate regression were entered into the multivariable regression. The cut-off point was defined as being statistically significant at 0.05. Variables that were not statistically significant but were clinically meaningful were retained in the multivariable model. The Hosmer-Lemeshow goodness-of-fit test was performed to check model adequacy. Analysis was performed using STATA version 13. Patients with missing data for AIS scores or RTS were omitted from the analysis and constituted less than 0.5 % of the study population. In keeping with the Utstein criteria, we excluded patients dead on arrival in hospital [21] with no response to resuscitation. Sensitivity analysis using imputation for missing RTS was performed using age, gender, and AIS scores, and for patients with missing AIS scores, imputation was performed using age, gender and RTS scores. There were no patients with both AIS and RTS scores missing in our registry.

Ethical approval was obtained from the first author’s Institutional Review Board, and all data in the NTR is de-identified, password-protected, and access limited to the premises of the National Registry of Diseases Office (NRDO).

## Results

There were 11,398 blunt trauma patients in our study, with 1114 patients meeting the anatomical criteria for polytrauma, and 1073 patients meeting either the physiological or the age criteria in the Berlin definition of polytrauma (Table 1). As with many ageing nations, the majority of our injuries are falls, followed by motor vehicle injuries. There were more males than females, and this was the case for all age groups up to age 75.

**Table 1:** Characteristics of national trauma registry patients aged over 18 (*n* = 11,398)^a^

	All	Age 18–44	Age 45–54	Age 55–64	Age 65–69	Age 70–74	Age 75–84	Age 85 and above
Study Population	11,398	3366	1260	1851	755	850	1982	1334
Male	7149 (62.7)	2788 (82.8)	1013 (80.4)	1223 (66.1)	442 (58.5)	444 (52.2)	836 (42.2)	403 (30.2)
Age mean (SD)	58.4 (21.6)	30.7 (7.5)	49.7 (2.9)	59.9 (2.9)	66.8 (1.5)	72.1 (1.4)	79.5 (2.9)	89.6 (3.9)
NISS mean (SD)	19.6 (12.8)	21.1 (12.5)	20.2 (12.7)	18.7 (13.1)	19.5 (13.3)	19.2 (12.9)	18.6 (13.0)	18.3 (12.5)
ISS mean (SD)	14.8 (8.1)	16.0 (9.3)	15.0 (8.3)	14.1 (8.0)	14.4 (7.8)	14.2 (7.3)	14.0 (7.0)	14.0 (6.7)
Polytrauma (Anatomical Definition)	1114 (9.8)	534 (15.9)	155 (12.3)	157 (8.5)	47 (6.2)	44 (5.2)	98 (4.9)	79 (5.9)
Polytrauma (Anatomical and one of: Systolic Blood Pressure (SBP) ≤ 90 mmHg or Glasgow Coma Scale (GCS) ≤ 8 or age 70 and above)	1073 (9.4)	512 (15.2)	142 (11.3)	151 (8.2)	47 (6.2)	44 (5.2)	98 (4.9)	79 (5.9)
Revised Trauma Score (RTS) mean (SD)	7.6 (0.9)	7.4 (1.1)	7.5 (1.0)	7.6 (0.8)	7.6 (0.7)	7.6 (0.8)	7.6 (0.6)	7.7 (0.6)
Systolic Blood Pressure (SBP) ≤ 90 mmHg	266 (2.3)	130 (3.9)	46 (3.7)	42 (2.3)	9 (1.2)	8 (0.9)	17 (0.9)	14 (1.0)
Glasgow Coma Scale (GCS) ≤ 8	758 (6.7)	311 (9.2)	102 (8.1)	119 (6.4)	36 (4.8)	44 (5.2)	85 (4.3)	61 (4.6)
Abbreviated Injury Score of 3 or more^b^								
Head and Neck	4975 (43.6)	1280 (38.0)	512 (40.6)	741 (40.0)	375 (49.7)	447 (52.6)	964 (48.6)	656 (49.2)
Face	94 (0.8)	62 (1.8)	10 (0.8)	12 (0.6)	5 (0.7)	2 (0.2)	3 (0.2)	0 (0.0)
Thoracic	2135 (18.7)	817 (24.3)	343 (24.2)	361 (19.5)	113 (15.0)	108 (12.7)	250 (12.6)	143 (10.7)
Abdominal	940 (8.2)	369 (11.0)	117 (9.3)	114 (6.2)	49 (6.5)	50 (5.9)	137 (6.9)	104 (7.8)
Extremity	3990 (35.0)	1183 (35.1)	395 (31.3)	749 (40.5)	240 (31.8)	265 (31.2)	689 (34.8)	469 (35.2)
External	10 (0.1)	7 (0.2)	1 (0.1)	2 (0.1)	0 (0.0)	0 (0.0)	0 (0.0)	0 (0.0)
Charlson Comorbidity Index (CCI)								
CCI 0	8285 (72.7)	3074 (91.3)	1053 (83.6)	1391 (75.1)	488 (64.4)	518 (60.9)	1061 (53.5)	700 (52.5)
CCI 1	1978 (17.4)	208 (6.2)	130 (10.3)	296 (16.0)	174 (23.0)	212 (24.9)	586 (29.6)	372 (27.9)
CCI 2	664 (5.8)	11 (0.3)	30 (2.4)	97 (5.2)	55 (7.3)	73 (8.6)	215 (10.8)	183 (13.7)
CCI 3	223 (2.0)	2 (0.1)	13 (1.0)	43 (2.3)	18 (2.4)	26 (3.1)	77 (3.9)	44 (3.3)
CCI of 4 and above	104 (0.9)	3 (0.1)	2 (0.2)	10 (0.5)	13 (1.7)	12 (1.4)	33 (1.7)	31 (2.3)
30-day Mortality	783 (6.9)	164 (4.9)	67 (5.3)	103 (5.6)	49 (6.5)	58 (6.8)	183 (9.2)	159 (11.9)
In-Hospital Mortality	167 (5.0)	72 (5.7)	116 (6.3)	54 (7.2)	62 (7.3)	183 (9.2)	149 (11.2)	803 (7.0)
Mechanism of Injury								
Motor Vehicle Injuries	3372 (29.6)	1,828 (54.3)	533 (42.3)	545 (29.4)	163 (21.6)	146 (17.2)	130 (6.6)	27 (2.0)
Falls	7166 (62.9)	1,023 (30.4)	595 (47.2)	1,194 (64.5)	562 (74.4)	674 (79.3)	1,824 (92.0)	1,294 (97.0)
Assault	212 (1.9)	132 (3.9)	36 (2.9)	22 (1.2)	7 (0.9)	8 (0.9)	7 (0.4)	0 (0.0)
Others	648 (5.7)	383 (11.4)	96 (7.6)	90 (4.9)	23 (3.1)	22 (2.6)	21 (1.1)	13 (1.0)

^a^Percentages in brackets (%) unless otherwise noted. SD = standard deviation

^b^% of study population, not adding up to 100 % due to patients with polytrauma

Parameter estimates and model adequacy measures from multivariable regression models were used to compare the ISS and NISS, with and without polytrauma. All models included age, gender, and Charlson comorbidity scores. The c-statistics for all models were greater than 0.8. The NISS and polytrauma models were superior to the ISS and polytrauma models, by at least one percentage point for all models. The results are reported in Table 2.

**Table 2:** ISS, NISS, polytrauma, and physiologic scores

Model	Variables (all include age, gender, and Charlson score)	OR for ISS or NISS (95 % CI)	OR for polytrauma, anatomical definition, yes/no (95 % CI)	OR for RTS (95 % CI)	C-statistic
1a	ISS	1.17 (1.16, 1.18) **	-	-	0.887
1b	NISS	1.11 (1.10, 1.11) **	-	-	0.902
2a	ISS + RTS	1.12 (1.11, 1.14)**	-	0.40 (0.37, 0.43)**	0.918
2b	NISS + RTS	1.08 (1.08, 1.09)**	-	0.44 (0.41, 0.48)**	0.926
3a	ISS + polytrauma	1.18 (1.17, 1.20)**	0.60 (0.46, 0.79)**	-	0.887
3b	NISS + polytrauma	1.10 (1.10, 1.11)**	2.48 (1.98, 3.11)**	-	0.904
4a	ISS + RTS + polytrauma	1.13 (1.12, 1.15)**	0.69 (0.51, 0.93)*	0.40 (0.37, 0.43)**	0.918
4b	NISS + RTS + polytrauma	1.08 (1.07, 1.09)**	1.98 (1.53, 2.54)**	0.45 (0.42, 0.49)**	0.928
5a	ISS + GCS + SBP + polytrauma	1.14 (1.12, 1.15)**	0.70 (0.52, 0.93)*	-	0.913
5b	NISS + GCS + SBP + polytrauma	1.08 (1.07, 1.09)**	1.98 (1.54, 2.54)**	-	0.924

*p < 0.05 **p < 0.001

*Abbreviations*: *CI* 95 % confidence interval, *GCS* Glasgow Coma Scale, *ISS* Injury Severity Score, *NISS* New Injury Severity Score, *OR* odds ratio, *RTS* Revised Trauma Score, *SBP* Systolic blood pressure

For models with ISS and polytrauma, the risk of mortality for patients with polytrauma was lower than those without polytrauma, with an odds ratio of 0.60. When used with the NISS, the risk of mortality for patients with polytrauma was higher than those without polytrauma, with an odds ratio of 2.48.

Physiological scores (GCS 8 or less and systolic blood pressure 90 mmHg or less, or RTS) increased the c-statistic for all the scores by at least 2 percentage points, with the GCS or systolic blood pressure categorical criteria being less than half a percentage point inferior to the RTS.

### Comparison for injuries in specific ISS body regions

To evaluate our hypothesis on the superiority of the NISS over the ISS for isolated head-injured patients compared to patients with dominant injuries in other ISS body regions, we performed the same analysis on the following subgroups where patients had an AIS score of 3 or more in the following ISS regions: head and neck, chest, abdomen, and extremity. There were too few patients to perform the analysis for facial injuries (due to the relatively low incidence of these injuries) and skin injuries (as we excluded isolated burns patients from our study). The summary of our findings are in Table 3.

**Table 3:** ISS, NISS, polytrauma, and physiologic scores, by sub-group analysis of ISS regions

ISS region (AIS score of 3 or more)	Model variables (all include age, gender, and Charlson score)	Polytrauma OR (95 % CI)	Poly-trauma *P*-value	C-statistic	Model variables (all include age, gender, and Charlson score)	Polytrauma OR (95 % CI)	Poly-trauma *P*-value	C-statistic
Head and Neck	ISS	-	-	0.849	NISS	-	-	0.877
	ISS + RTS	-	-	0.898	NISS + RTS	-	-	0.915
	ISS + Polytrauma	0.62 (0.46, 0.85)	0.003	0.850	NISS + Polytrauma	2.49 (1.92, 3.25)	<0.001	0.882
	ISS + Polytrauma + RTS	0.63 (0.44, 0.89)	0.008	0.899	NISS + Polytrauma + RTS	1.83 (1.36, 2.47)	<0.001	0.916
Chest	ISS	-	-	0.901	NISS	-	-	0.929
	ISS + RTS	-	-	0.935	NISS + RTS	-	-	0.941
	ISS + Polytrauma	1.64 (0.95, 2.83)	0.077	0.900	NISS + Polytrauma	2.22 (1.36, 3.60)	0.001	0.915
	ISS + Polytrauma + RTS	1.35 (0.74, 2.44)	0.325	0.934	NISS + Polytrauma + RTS	1.66 (0.97, 2.82)	0.062	0.941
Abdomen	ISS	-	-	0.926	NISS	-	-	0.933
	ISS + RTS	-	-	0.949	NISS + RTS	-	-	0.954
	ISS + Polytrauma	2.24 (0.68, 7.4)	0.186	0.929	NISS + Polytrauma	3.43 (1.05, 11.28)	0.042	0.951
	ISS + Polytrauma + RTS	2.52 (0.73, 8.71)	0.143	0.948	NISS + Polytrauma + RTS	1.62 (0.89, 2.94)	0.111	0.958
Extremity	ISS	-		0.886	NISS	-		0.870
	ISS + RTS	-		0.900	NISS + RTS	-		0.891
	ISS + Polytrauma	0.99 (0.47, 2.09)	0.98	0.886	NISS + Polytrauma	2.02 (1.08, 3.80)	0.028	0.870
	ISS + Polytrauma + RTS	1.14 (0.52, 2.48)	0.72	0.900	NISS + Polytrauma + RTS	2.02 (1.02, 4.01)	0.044	0.891

*Abbreviations*: *AIS* Abbreviated Injury Scale, *CI* 95 % confidence interval, *ISS* Injury Severity Score, *NISS* New Injury Severity Score, *OR* odds ratio, *RTS* Revised Trauma Score

The c-statistic for patients with severe injuries in the head and neck region was more than two percentage points higher for the NISS compared to the ISS, and three percentage points higher for ‘NISS and polytrauma’ compared to ‘ISS and polytrauma’. Adding polytrauma to the NISS for these patients improved the c-statistic by half a percentage point.

With the addition of physiological variables, the improvement of prediction was four percentage points for ‘NISS and polytrauma’ , while it was five percentage points for ‘ISS and polytrauma’. While our registry has a low proportion of missing physiological variables, this has important implications for registries with incomplete physiological variables where real-time initial physiological data is missing.

For other subgroups of injuries, the difference in c-statistic was no higher than two percentage points, with ‘NISS and polytrauma’ being superior for all groups, except for extremity injuries where the ISS models were superior by up to two percentage points.

We also looked at the combination of NISS and the number of injuries, as proposed by some authors [38]. The c-statistic for this model was similar to the ‘NISS and polytrauma’. The highest number of injuries documented for a single patient in our registry was 51, which would have required fairly exhaustive documentation.

When the RTS was replaced by GCS of 8 or less and systolic blood pressure of 90 mmHg or less, similar trends in c-statistic were achieved, which suggests that, where the exact physiological data is incomplete, inferring these values from clinical case records is almost as useful as the RTS in outcome prediction.

Sensitivity analyses included adjusting for age as linear variable, race, residency status, gender, age by gender interaction, mechanism of injury, and replacing the Charlson Comorbidity Index with the top 5 % of actual comorbidities in our study population (diabetes, hypertension, hyperlipidaemia and cancer) [3]. None of these altered our findings.

### Final Models

Our final model of choice (NISS, polytrauma and RTS) is presented in Table 4. When the RTS was replaced by GCS of 8 or less and systolic blood pressure of 90 mmHg or less, similar trends in c-statistic were achieved, which suggests that, where the exact physiological data is incomplete, infer- ring these values from clinical case records is almost as useful as the RTS in outcome prediction (Table 5).

We also looked at the combination of NISS and the number of injuries, as proposed by some authors [38]. The c-statistic for this model was similar to the ‘NISS and polytrauma’. The highest number of injur- ies documented for a single patient in our registry was 51, which would have required fairly exhaustive documentation.

Sensitivity analyses included adjusting for age as linear variable, race, residency status, gender, age by gender interaction, mechanism of injury, and replacing the Charlson Comorbidity Index with the top 5 % of actual comorbidities in our study population (diabetes, hyper- tension, hyperlipidaemia and cancer) [3]. None of these altered our findings.

## Discussion

In our study, we found that models using the NISS and polytrauma together outperformed models using the ISS and polytrauma together, NISS alone, or ISS alone in predicting mortality of patients who had moderate to severe trauma. We found that incorporating the categorical variables for GCS 8 or less and systolic blood pressure of 90 mmHg or less increased the prediction of the model, with a c-statistic greater than 0.90.

As hypothesized, the NISS underestimated the risk of death for patients with polytrauma. Furthermore, the ISS overestimated the risk of death for patients with polytrauma as they have a lower risk of death than patients without polytrauma when the ISS was used as the anatomical injury severity score. This is likely due to the fact that the way ISS is calculated already incorporates a measure of polytrauma in its score, rather than a true reflection of reduced mortality in this group of patients.

One criticism of using the ISS alone for institutional benchmarking is the potential underestimation of mortality for patients with isolated head injuries [17]. These patients usually have a poor prognosis independent of the treatment rendered, hence explaining the superior c-statistic for some of the scoring systems that give separate or increased weight to head injuries [39, 40]. Physiological scores like the Revised Trauma Score (RTS) that incorporate the Glasgow Coma Scale (GCS) can enhance outcome prediction in these patients [31]. However, incomplete physiological data, especially in the patients with worse prognoses, is a problem, and patients with missing physiological scores have been shown to have worse outcomes compared to similarly injured patients without missing physiological data [41]. More complex scoring systems have been developed specifically for head injured patients [42–44]. These specialised head-injury scores require additional detailed radiological and clinical information not always captured by trauma registries.

It has been suggested that the NISS is superior to the ISS for head injuries [45, 46] and that the score discrepancy (i.e. NISS higher than ISS) for non-survivors is greatest for head-injured patients. Clinically, it would be expected that each individual AIS score in the head region would have a cumulative effect on mortality, whereas the ISS score would only reflect the contribution of the maximum of these injuries. As such, study populations with high proportions of head-injured patients would find the NISS superior to ISS. Some authors have proposed a separate variable for head injury in their trauma prediction model [39]. However, a score that is good at predicting outcomes for head-injured patients can under-perform for head-injured patients with polytrauma [47]. This would explain why our model incorporating NISS and polytrauma improves prediction for this group of patients.

In our study, the NISS outperformed the ISS for head-injured patients, both when used in alone and when combined with the polytrauma variable. More importantly, the ‘NISS and polytrauma’ model yielded a good c-statistic for this region even without physiological variables, which is useful in cases where physiological data on arrival is incompletely captured. The c-statistic for our model for head-injured patients compares favourably to the complex head-injury-specific scoring systems that require complex radiological, clinical, and treatment data [41, 43, 48]. It also compares favourably to the revised injury severity classification score (RISC) that was recently validated specifically for head-injured patients [39, 47] and which achieves a c-statistic in excess of 0.90 for all-trauma patients, incorporating additional parameters such as biochemical markers of injury severity (acidosis, coagulation, haemoglobin), pupil size and reaction, and cardiopulmonary resuscitation [39]. However, such extensive data collection may not be possible in all institutions or systems.

Head-injury-specific scoring systems are important for comparing outcomes for head-injured patients in dedicated neuro-trauma units with the resources to collect the necessary data. However, for comparison of outcomes across multiple study populations and case-mix of blunt trauma, our model performs favourably relative to the other scoring systems.

There was little difference between the ISS and NISS for patients with chest and abdominal injuries, and the NISS was slightly worse than the ISS for extremity injuries. However, while our main outcome variable was in-hospital mortality, some authors have found the NISS to be superior to the ISS for predicting an extended length of stay and intensive care admission for patients with musculo-skeletal injuries [15], as well as in predicting mortality, sepsis, and multi-organ failure [11].

When used together with the NISS and polytrauma, we found that the physiological information in the two categorical variables (systolic blood pressure of 90 mmHg or less and GCS of 8 or less) outperformed the ‘ISS and RTS’ model. This could be useful in addressing the problem of missing RTS values in large registries. For example, for critically ill patients where the blood pressure is recorded as “unrecordable”, or patients that require intubation due to a “low GCS”, these patients would like have a “missing” RTS, but it could be inferred that they would meet have a systolic blood pressure of 90 mmHg or less and GCS of 8 or less respectively [21].

The goal of our study was to show the effect of adding the polytrauma term to the NISS score in improving prediction of mortality after trauma. In our model, we used the Charlson Comorbidity Score as the main covariate of comorbidity. A European national registry study achieved a c-statistic of more than 0.95 incorporating the NISS, Triage Revised Trauma Score, age, and American Society of Anesthesiologists Physical Status Classification System (ASA-PS) [49]. While the ASA-PS may a better indicator of comorbidity for trauma patients undergoing surgery, it was not available in our registry and depends on the accuracy of the duty anaesthetist’s assessment of the patient at the time of surgery. Hence, we suggest that the NISS and polytrauma model replace the NISS or the ISS alone as the main benchmark of anatomical severity of injury, regardless of how other important predictors of survival (age, comorbidities, physiology) are measured.

The novelty of our paper is the incorporation of the newly-defined polytrauma definition into the existing NISS score to improve mortality after injury regardless of population casemix. One of the limitations of our study is that we were unable to examine the biochemical parameters (acidosis and coagulopathy) proposed in the Berlin definition of Polytrauma as these were not routinely collected by the Singapore NTR at the time of study. In addition, our exclusion of minor injuries with an ISS of less than 9 meant that we could not test our model on minor injuries, although this group of patients would be expected to have a low trauma-related mortality. Also, our population does not have enough penetrating injury to be able to test this model on penetrating trauma.

There are some limitations due to the low numbers of polytrauma patients in our study. Firstly, we were not able to test our model with a separate construction and validation dataset. Therefore, validation will have to be undertaken in a separate analysis using another database. Secondly, this may reflect a higher proportion of low-velocity injuries in our database overall, and hence may not apply to settings where the majority of patients are high-velocity injuries. Finally, with the low proportion of polytrauma patients, there is a possibility that our model findings could be due to differences in optimal treatment received by polytrauma patients compared to non-polytrauma patients. For example, this could be the case if our trauma system salvages fewer polytrauma patients due to poorer co-ordination of care compared to trauma systems who routinely treat a higher proportion of polytrauma patients, although the reduced risk of death in polytrauma patients in the ISS model suggests that this is not the case. We hope that studies from other registries can address this limitation.

## Conclusions

Adding polytrauma to the NISS or ISS improves prediction of 30-day mortality. The superiority of the NISS or ISS depends on the proportion of polytrauma and head-injured patients in the study population. The ISS overestimates mortality in polytrauma patients due to the nature of how it is calculated, while the NISS underestimates it. Incorporating both NISS and polytrauma into our prediction model addresses the shortcomings of both of these commonly-used AIS-based injury scoring systems.

The NISS and polytrauma model outperformed ISS and NISS alone, without increasing the amount of data required or complexity of scoring, particularly for the sub-group of head-injured patients.

When combined with dichotomous categorical variables comprising the physiological information used in the Berlin definition of polytrauma (GCS of 8 or less, or systolic blood pressure of 90 mmHg or less), the NISS and polytrauma model outperformed models based on the ISS and RTS, which is useful for addressing the problem of missing data for severely ill patients.

### Ethics approval and consent to participate

The first author’s (Singapore General Hospital) Institutional Review Board granted ethical approval for this retrospective study, as required prior to gaining access to the NTR data, which is de-identified prior to release for research, password-protected and access limited to the premises of the National Registry of Diseases Office (NRDO). Consent was not obtained because information was anonymized and de-identified prior to analysis, as per NRDO protocol.

### Consent for publication

Not applicable.

### Availability of data and materials

The data was obtained from a third party, the National Trauma Registry, established by Singapore’s Ministry of Health (MOH). Data are available from the National Registry of Diseases Office in Singapore for researchers who meet the criteria for access to confidential data. Details are available at https://www.nrdo.gov.sg/data-request.

## Appendices

The New Injury Severity Score (NISS) + Polytrauma + Physiology model for prediction of mortality after trauma.

### Appendix 1

**Table 4:** New Injury Severity Score (NISS) + Polytrauma + Revised Trauma Score (RTS)

Variable	Category	Coefficient	OR (95 % CI)	*P*-value
New Injury Severity Score (NISS)		0.08	1.08 (1.07, 1.09)	<0.001
Polytrauma (reference no polytrauma)		0.68	1.98 (1.53, 2.54)	<0.001
Revised Trauma Score (RTS)		−0.80	0.45 (0.42, 0.49)	<0.001
Age	18–44		1 (reference)	
	45–54	0.25	1.29 (0.82–2.01)	0.272
	55–64	0.94	2.56 (1.77–3.71)	<0.001
	65–69	1.36	3.90 (2.46–6.18)	<0.001
	70–74	1.40	4.04 (2.57–6.34)	<0.001
	75–84	2.07	7.95 (5.58–11.32)	<0.001
	85 and above	2.58	13.26 (9.13–19.25)	<0.001
Gender (reference female)		0.15	1.17 (0.94,1.45)	0.167
Charlson Comorbidity index	0		1 (reference)	
	1	0.04	1.04 (0.80–1.35)	0.786
	2	0.62	1.86 (1.33–2.60)	<0.001
	3	1.20	3.31 (2.03–5.38)	<0.001
	4 or more	1.24	3.47 (1.85–6.51)	<0.001
Constant		−0.72	0.49 (0.25–0.95)	0.034

*Abbreviations*: *CI* 95 % confidence interval, *OR* odds ratio, *NISS* New Injury Severity Score, *RTS* Revised Trauma Score

### Appendix 2

**Table 5:** New Injury Severity Score (NISS) + Polytrauma + Systolic Blood Pressure (SBP) + Glasgow Coma Scale (GCS)

Variable	Category	Coefficient	OR (95 % CI)	*P*-value
New Injury Severity Score (NISS)		0.08	1.08 (1.08–1.09)	<0.001
Polytrauma (reference no polytrauma)		0.68	1.98 (1.54–2.54)	<0.001
SBP 90 mmHg or less (reference SBP 90 mmHg and above)		−1.25	0.29 (0.19–0.43)	<0.001
GCS 8 or less (reference GCS more than 8)		−2.11	0.12 (0.09–0.16)	<0.001
Age	18–44		1 (reference)	
	45–54	0.19	1.21 (0.79–1.85)	0.376
	55–64	0.76	2.14 (1.50–3.05)	<0.001
	65–69	1.21	3.37 (2.15–5.27)	<0.001
	70–74	1.26	3.52 (2.27–5.46)	<0.001
	75–84	1.95	7.05 (5.00–9.93)	<0.001
	85 and above	2.44	11.48 (8.00–16.48)	<0.001
Gender (reference female)		0.17	1.19 (0.95–1.47)	0.126
Charlson Comorbidity index	0		1 (reference)	
	1	0.02	1.03 (0.79–1.33)	0.852
	2	0.62	1.85 (1.32–2.60)	<0.001
	3	1.23	3.42 (2.10–5.55)	<0.001
	4 or more	1.29	3.64 (1.95–6.79)	<0.001
Constant		−3.49	0.03 (0.02–0.05)	<0.001

*Abbreviations*: *CI* 95 % confidence interval, *GCS* Glasgow Coma Scale, *OR* odds ratio, *SBP* Systolic blood pressure

## Abbreviations

AIS: abbreviated injury scale
CI: 95 % confidence interval
GCS: Glasgow coma scale
ISS: injury severity score
NISS: new injury severity score
NTR: National Trauma Registry (Singapore)
OR: odds ratio
RISC: revised injury severity classification
RTS: revised trauma score
SBP: systolic blood pressure
SCDF: Singapore Civil Defence Force (ambulance)
SD: standard deviation
